# RETURN MIGRATION AND AGING OF JAPANESE INDIVIDUALS LIVING IN GREATER NEW YORK: A QUANTITATIVE ANALYSIS

**DOI:** 10.1093/geroni/igac059.1831

**Published:** 2022-12-20

**Authors:** Itsuko Toyama, Taeko Nakashima

**Affiliations:** St. Andrew's University, Izumi City, Osaka, Japan; Nihon Fukushi University, Handa-city, Aichi, Japan

## Abstract

Our previous quantitative research studies have revealed that large numbers of older Japanese individuals living in overseas communities either maintain hope of receiving culture-oriented care or harbor aspirations of returning to Japan to live out their final days. Presently, by analyzing a quantitative research study on older Japanese individuals living in Greater New York in 2018, we clarify the characteristics of those individuals who have decided to return to Japan. In the research study, 2,057 questionnaires were distributed, and the overall return rate was 29.7%. Respondents were divided into three groups based on their answers regarding their intentions for the location of their final abode: the United States, Japan, or indecisive. We performed a multinomial logistic regression analysis based on age, gender, immigration status, having children in the United States, and academic background. The following is a summary of the findings: (1) as participants aged, the ratio of selecting either “Japan” or “indecisive” as opposed to “the United States” decreased, (2) compared to those holding citizenship, individuals with permanent residence selected both “Japan” and “indecisive” at greater rates (by approximately 3 times and 8.7 times, respectively); (3) compared to those having children in the United States, individuals without children in the United States selected both “Japan” and “indecisive” at greater rates (by approximately 1.8 times and 2.2 times, respectively); (4) individuals with a graduate career displayed a tendency to select “the United States,” and (5) gender and language proficiency were not critical attributes in determining the likelihood of selecting “Japan.”

